# Evidence for Changes in Screen Use in the United States During Early Childhood Related to COVID-19 Pandemic Parent Stressors: Repeated Cross-Sectional Study

**DOI:** 10.2196/43315

**Published:** 2024-05-22

**Authors:** Jill Glassman, Kathryn L Humphreys, Adam Jauregui, Arnold Milstein, Lee Sanders

**Affiliations:** 1 Clinical Excellence Research Center (CERC) Stanford University School of Medicine Stanford, CA United States; 2 Department of Psychology and Human Development Vanderbilt University Nashville, TN United States; 3 Oracle Life Sciences Austin, TX United States; 4 Departments of Pediatrics Stanford University Stanford, CA United States

**Keywords:** child health, parent-child relationship, screen time, technoference, health equity

## Abstract

**Background:**

The COVID-19 pandemic transformed the home lives of many families in the United States, especially those with young children. Understanding the relationship between child and parent screen time and family stressors exacerbated by the pandemic may help inform interventions that aim to support early child development.

**Objective:**

We aim to assess the changing relationship between family screen time and factors related to pandemic-induced remote work and childcare or school closures.

**Methods:**

In the spring of 2021 we administered a survey, similar to one administered in the spring of 2019, to a national sample of parents of young children (aged 6 to 60 months). Using iterative sampling with propensity scores, we recruited participants whose sociodemographic characteristics matched the 2019 survey. Participants were aged >18 years, proficient in English or Spanish, and residing in the United States. The main outcomes were changes in child screen time (eg, mobile phone, tablet, computer, and television) and parenting technoference, defined as perceived screen-related interference with parent-child interactions. Additional survey items reported pandemic-related job loss, and changes to work hours, work location, caregiving responsibilities, day care or school access, and family health and socioeconomic status.

**Results:**

We enrolled 280 parents, from diverse backgrounds. Parents reported pandemic-related changes in child screen time (mean increase of 1.1, SD 0.9 hours), and greater parenting technoference (3.0 to 3.4 devices interfering per day; *P=*.01). Increased child screen time and parenting technoference were highest for parents experiencing job loss (mean change in child screen time 1.46, SD 1.03; mean parenting technoference score 3.89, SD 2.05), second highest for working parents who did not lose their job (mean change in child screen time 1.02, SD 0.83; mean parenting technoference score 3.37, SD 1.94), and lowest for nonworking parents (mean change in child screen time 0.68, SD 0.66; mean parenting technoference score 2.66, SD 1.70), with differences significant at *P*<.01. School closure and job loss were most associated with increased child screen time during the pandemic after controlling for other stressors and sociodemographic characteristics (*d*=0.52, *P<*.001; *d*=0.31, *P*=.01). Increased child screen time and school closure were most associated with increased parenting technoference (*d*=0.78, *P<*.001; *d*=0.30, *P*=.01).

**Conclusions:**

Work and school changes due to the COVID-19 pandemic were associated with increased technology interference in the lives of young children. This study adds to our understanding of the interaction between technology use at home and social factors that are necessary to support early childhood health and development. It also supports possible enhanced recommendations for primary care providers and childcare educators to guide parents in establishing home-based “screen time rules” not only for their children but also for themselves.

## Introduction

Use of electronic technologies use may be an important determinant of maternal and child health. Information technology, including its corollary “screen time,” has intruded into the everyday lives of the youngest children and the newest parents [1]. Since they became increasingly essential tools to facilitate remote connections, learning, and entertainment during the COVID-19 pandemic, the use of screen-enabled technologies (eg, smartphones, tablets, and laptop computers) increased for both young children and their parents [2-4].

Though not all technology use is detrimental to child health [5-7], increased screen use by young children can interfere with parent-child interactions [8,9]. For infants and young children, their primary interaction partners are their adult caregivers, typically parents. Ecodevelopmental theory proposes that contextual features of a child’s environment, including aspects that disrupt their caregiving experiences, are likely to influence socioemotional and cognitive development. Indeed, parents’ own use of mobile and other technological devices (termed “parenting technoference”) is associated with early childhood problem behaviors, delayed language acquisition, and poor healthy eating habits [10-13]. In particular, studies show that parent use of mobile devices interferes with their ability to respond to child cues and bids for attention [13-15]. This phenomenon was made even more complex during the pandemic when parent or child screen time increased for a variety of reasons [2,16,17].

Parent stress—which increased during the pandemic for multiple reasons, including job loss and related difficulties from lost income, expectations to work full-time from home while simultaneously caring for children, and managing children’s schooling at home—may have further increased both child screen time [10] and parenting technoference [15,18]. Examining how the screen time of parents and their young children changed during the pandemic, especially for pandemic-related stressors, may provide useful insights into how mobile technology use by both young children and their caregivers is changing parent-child interactions and child development. This in turn is critical to informing policies, programs, and parents with meaningful guidance regarding their use [13,19].

In this study, we aimed to examine the research question, “How did child and parent screen use and home-based ‘parenting technoference’ in the United States change since the COVID-19 pandemic, and how were these changes moderated by pandemic-related socioeconomic stressors?” To accomplish this, we conducted a national survey of parents of young children (aged 6 months to 5 years) during the pandemic’s first year, modeled on a similar survey administered before the pandemic [20]. The previous survey’s parent or child screen time measurement aim overlapped with this study’s surveys, while other aims (eg, the relationship between parent screen time and parent desire for help to reduce screen time) did not [20]. We hypothesized that the pandemic would be associated with increases in outcome variables measuring child or parent screen use and technoscience. We also hypothesized that increases in these screen time and technoference outcomes would be most pronounced among families that encounter more socioeconomic stressors (eg, job loss, food insecurity, and family dysfunction). To our knowledge, the unique interrelationship between parenting technoference, child screen time, and pandemic-induced remote work and reduced childcare has not been studied in a representative US sample. This is especially relevant to emerging issues regarding dual responsibilities of childcare supervision while simultaneously working for pay.

## Methods

### Study Design

In March and April of 2021, we conducted an observational repeated cross-sectional study by administering a population-based web-based survey of US parents of children younger than 5 years previously administered in May and June of 2019 [20]. Recruitment and administration of this open, voluntary, web-based survey were performed using CloudResearch (TurkPrime; Prime Research Solutions LLC) and Qualtrics (Qualtrics). As in the 2019 survey, participants could choose to take the survey in English or Spanish (details of translation in Glassman et al [20]). Participants were provided reminders for unanswered questions and were able to review responses via a back button before submission.

### Ethical Considerations

All participants were provided an informed consent form, discussing survey length and anonymity of responses, before being taken to the first survey question. All procedures were approved by Stanford’s Human Subjects Research Office (institutional review board protocol 57720).

### Participants

Inclusion criteria were adults aged >18 years with primary caregiving responsibility for at least 1 child aged >6 months and <5 years in the household. Exclusion criteria were the inability to read English or Spanish or completing the survey outside of the United States. For the survey, the index child was defined as the youngest child in the household. We sought a sample of participants whose sociodemographic characteristics matched that of the 280 participants in the 2019 survey. To accomplish this, CloudResearch oversampled participants, and we used iterative, propensity score matching until we were able to create a matched sample. As noted in the Limitations section, the present study’s sample therefore carried the same generalizability strengths and weaknesses as the sample obtained for the 2019 survey [20]. For example, we aimed to match how participants in the original survey were distributed across the 4 census regions of the United States (Midwest: 52/280, 19%; Northeast: 54/280, 19%; South: 119/280, 42%; and West: 55/280, 20%).

### Survey Items

We used the same survey items about parent perceptions of their own technology use, parent perceptions of their technology use in the presence of their young child (parenting technoference), and sociodemographic factors, as described in detail in the publication of the 2019 survey results [20]. Additionally, we added items that assessed parents’ perceptions of changes in their child’s average daily screen time, as well as items capturing pandemic-related stressors. Wording for all items and construct coding is provided in detail in the web-based supplementary material and summarized (see Multimedia Appendix 1). The survey was translated into Spanish by a trained, bilingual research associate and back-translated from Spanish to English by another independent, trained bilingual research associate; differences between the original and back-translated versions were resolved in Spanish by a third bilingual research coordinator.

### Outcome Measures

The primary outcomes were (1) the extent to which parents perceived their child’s screen time to have increased since the pandemic began, (2) the extent to which parents perceived their mobile device use as interfering in their interactions with their young children [21], and (3) parents’ perceptions of the degree to which their own mobile device use was problematic in general (eg, reported inability to resist checking text messages) [21]. Outcome 1 (change in child screen time) was assessed by asking how many more hours per day the index child used each of the following 6 devices: television, computer, smartphone, tablet, other handheld device (eg, iPod Touch), and video game console. Response options were 0, <1, 1, 2, 3, 4+ more hours per day and were coded as 0, 0.5, 1, 2, 3 and 4, respectively. Outcome 2 was measured using the parenting technoference index, which is a count from 0 to 6 of the number of devices (eg, smartphone and tablet) that interrupted a conversation or activity between the parent and index child at least one time on a typical day [18]. Outcome 3 was assessed using the parent problem technology use scale, which is an average of responses to 3 questions such as “when my mobile electronic device alerts me to indicate new messages, I cannot resist checking them,” with 6-point Likert scale response options ranging from strongly disagree to strongly agree [18].

### Measures of Pandemic-Related Stressors

Independent variables representing pandemic-related stress were measured using new and previously validated items and scales assessing whether parents experienced the following potential stressors during the first year of the pandemic: job loss (yes, no, or did not have a job before the pandemic), reduction in job hours (yes, no, or not applicable [N/A]), change to remote work (yes, no, or N/A), child day care or preschool or school closure (yes, no, or N/A), change in caregiving time (decreased, increase by 1, 2, 3, 4+ hours, coded as –1 to 4), and reduced ability to meet family health and socioeconomic needs (3-point scale from less difficult to more difficult across 4 domains—health care, food, utilities, and housing). We also examined how the pandemic-related increase in child screen time outcome was related to the technoference and parent problem technology use outcomes.

### Measures of Sociodemographic and Household Characteristics

The sociodemographic and household characteristics were selected because they have been hypothesized to be confounding variables in similar studies of child and parent screen time. They included self-report of age (parent and child), sex, race or ethnicity, language spoken at home, marital status, number of children, education level, and income level. A proxy measure of the geographic region in which the participant resided was derived from the longitude or latitude values of the survey respondent’s computer captured by CloudResearch survey administration. Geographic region was categorized into the 4 US census regions defined by West, South, Northeast, and Midwest.

### Data Analysis

We first conducted data distribution and quality assessments to identify potential missing values and outliers. We removed outliers defined as “speeders” (those who answer unreasonably fast) and “straightliners” (those who answer with identical values for each survey item in a block) as described for the 2019 survey [20,22,23]. All returned surveys were analyzed.

### Propensity Score Matching Procedures

To obtain a 2021 survey sample that matched our 2019 survey sample as closely as possible on the measured covariates, we used optimal pair full matching, which attempts to pair each “treatment” unit—in our case, the observations from the 2021 survey—with one or more “control” units—the observations from the 2019 survey. To accomplish this, a propensity score was estimated for each observation in the 2019 and 2021 survey using a logistic regression of the observation’s “treatment” status on the following covariates: parent age, sex, Hispanic ethnicity, language spoken in the home, number of children, education, income, geographic region, and marital status. Each observation from the 2019 survey was then paired with an observation from the 2021 survey such that the sum of the differences between propensity scores across the pairs was minimized. The 2021 survey observations satisfying this criterion were selected as the matched sample. Following this procedure, the *t* tests (2-tailed tests) and chi-square tests used to validate that the differences between the samples on each of the covariates were not significant. We used the *MatchIt* package in R (R Foundation for Statistical Computing) to accomplish the match [24].

### Bivariate Analyses

To assess whether parenting technoference and parent problem technology use was higher during the pandemic than in 2019, we used 2-sample *t* tests and Wilcoxon rank sum tests, given that our sample size was only moderate [25]. We report results from the *t* tests since the results were virtually identical.

For the 2021 survey results, the bivariate association between each outcome and pandemic-related stressor or sociodemographic characteristic was assessed using *t* tests. The results of these tests were used to screen for variables to enter into multivariable regression models given the relatively small sample size. We used a stricter screening criterion (*P*.05) than in our prior study because we had a larger pool of potential independent variables and covariates to assess in this study. We did not include the job change (to remote) or job hours reduced variables in multivariable models given they were missing for all parents who were not working before the pandemic. We also elected to include the education and not income variables given their high correlation.

### Multivariable Regression Analyses

To evaluate the independent association between the outcomes and pandemic-related stress and sociodemographic characteristics we estimated linear regression models. Separate models were used for each outcome. We assessed the association between a given independent variable and outcome by examining both the statistical significance (at *P*.05) of the Wald test for its regression coefficient as well as a measure of effect size. The effect size was estimated using Cohen *d* and partial η^2^. All analyses were conducted using the R (version 3.5.3; R Core Team).

## Results

### Sample Characteristics and Propensity Score Matching

To obtain a sample of 280 parents of children aged 0-5 years matching the sociodemographic characteristics of the prepandemic sample, 517 consenting participants meeting eligibility criteria were recruited in stages between March and April of 2021. Of these, 468 participants met the data quality criteria. The *MatchIt* algorithm in R requires that there are no missing covariate values, which resulted in a pool of 443 observations for matching. Table 1 shows that the optimal pair matching algorithm was able to select 280 observations from this sample such that all covariate means or percentage distributions matched within 1 point, and there were no statistically significant differences in characteristics between the 2 groups (*P*>.70 for each characteristic). The mean age of respondents was 33 (SD 8) years, with 80% (223/280) female and 20% (57/280) male, 68% (192/280) White, 12% (33/280) Black, and 24% (67/280) Hispanic participants. Almost half (133/280, 48%) had at least a college degree, with 24% (66/280) of them reporting some college, and 29% (81/280) of them having a high school degree or lower educational attainment.

**Table 1:** Descriptive statistics for study samples (N=280).

Characteristic		First parent survey (spring 2019)	COVID-19 survey (spring 2021)	*P* value^a^	
Age (years), mean (SD)		32.8 (8.4)	32.9 (9.5)	.95	
**Sex, n (%^b^)**					.99
	Female	222 (79)	223 (80)		
	Male	57 (20)	57 (20)		
	Other	1 (1)	—^c^		
**Race, n (%)**					N/A^d^ (items differed)
	Asian	16 (6)	30 (11)		
	Black	24 (9)	33 (12)		
	Hispanic	40 (15)	—		
	Mixed	—	5 (2)		
	Other	6 (2)	20 (7)		
	White	184 (68)	192 (68)		
**Hispanic or Latino, n (%)**					.70
	Yes	72 (26)	67 (24)		
	No	208 (74)	213 (76)		
**Language spoken at home, n (%)**					.99
	English	241 (86)	241 (86)		
	Other	39 (14)	39 (14)		
**Number of children, n (%)**					>.99
	1	108 (39)	108 (39)		
	>1	172 (61)	172 (61)		
**Education, n (%)**					.99
	≤HS^e^	81 (29)	81 (29)		
	Some college	65 (23)	66 (24)		
	≥College degree	134 (48)	133 (48)		
**Income (US $), n (%)**					.99
	<25,000	57 (20)	55 (20)		
	25,000 to <49,999	68 (24)	68 (24)		
	50,000 to <74,999	65 (23)	68 (23)		
	75,000 to <99,999	49 (18)	48 (18)		
	>100,000	41 (15)	41 (15)		
**Geographic area, n (%)**					.91
	Midwest	57 (20)	52 (19)		
	Northeast	49 (18)	54 (19)		
	South	117 (42)	119 (43)		
	West	57 (20)	55 (20)		
**Marital status, n (%)**					.84
	Single	62 (22.1)	65 (23)		
	Not single	218 (77.9)	215 (77)		

^a^*P* value for 2-sided independent samples *t* test.

^b^Percentages add to slightly <100 in some cases due to rounding.

^c^Not available.

^d^N/A: not applicable.

^e^HS: high school.

### Bivariate Results: Changes in Screen Time Outcomes

Table 2 shows that while parent reports of their absolute mean level of screen time did not change, mean levels of parents’ perceptions of their own parenting technoference and problem device use were higher in the spring 2021 midpandemic sample than the spring 2019 prepandemic sample (3.0, SD 2.1, vs 3.4, SD 2.0; 3.7, SD 1.3, vs 4.0, SD 1.2). Differences for each outcome were statistically significant (*P*<.05). The mean for the change in child screen time variable was 1.1 (SD 0.90) on a scale of 0 to 4, where 0 represented no change, and 4 represented an increase of 4 or more hours.

**Table 2:** Comparison of outcomes before and during COVID-19.

Outcome (scale)	Pre–COVID-19 pandemic (spring 2019), mean (SD)	During the COVID-19 pandemic (spring 2021), mean (SD)	*P* value^a^
Parenting technoference (0-6)	3.0 (2.1)	3.4 (2.0)	.01
Problem technology use (0-6)	3.7 (1.3)	4.0 (1.2)	.03
Change in child screen time since pandemic^b^ (0-4)	Not asked	1.1 (0.90)	N/A^c^

^a^*P* value for 2-sample *t* test.

^b^Child screen time was not asked in the pre–COVID-19 survey.

^c^N/A: not applicable.

### Bivariate Results: Association Between Sociodemographic and Pandemic-Related Stressors and Outcomes

Table 3 shows the results of bivariate analyses of the relationships between the outcomes and the sociodemographic and pandemic-related stress variables. Male caregivers (eg, fathers) on average reported higher levels of increased child screen time (1.45, SD 1.07, vs 1.01, SD 0.83; *P*=.01) and higher levels of their own mobile technology use interfering with interactions with their young child (3.91, SD 1.97, vs 3.32, SD 1.97, *P=*.05) since the pandemic. Reports of parenting technoference were greater, on average, for those with a college degree or higher than for those with some college or no college (3.82, SD 2.02, vs 3.21, SD 1.83, and 3.0, SD 1.92, respectively; *P=*.01), and for those with a family income above US $75,000 than for those with an income less than US $75,000 (4.04, SD 1.92, vs 3.16, SD 1.95, *P*<.001). Reports of increase in child screen time and parenting technoference were highest for working parents who lost a job during the pandemic (1.46 and 3.89, respectively), second highest for working parents who did not report losing their job during the first year of the pandemic (1.02 and 3.37, respectively), and lowest for nonworking parents (0.68 and 2.66, respectively), with differences significant at *P=*.01. Among working parents, those whose job changed to remote reported higher levels of pandemic-related increases in child screen time (1.35 vs 1.02; *P=*.01) and parenting technoference (4.18 vs 3.19; *P*<.001) than those whose did not. Reports of increases in parenting technoference and child screen time were higher among parents of children whose day care or preschool or school closed during the pandemic than among those whose child’s day care or preschool or school did not close and those whose child did not attend school (4.07 vs 3.29 and 2.65, respectively, *P*<.001; 1.40 vs 0.96 and 0.75; *P=*.002). Change in child screen time was moderately and statistically significantly correlated with parent technoference (*r*=0.44, *P*<.001).

**Table 3:** Bivariate associations between outcomes and sociodemographic and COVID-19–related family stress measures.

		Outcome		
Sociodemographic or COVID-19–related stressor		Change in child screen time since pandemic (0-4)	Parenting technoference (0-6)	Problem technology use (0-6)
**Age (parent)**				
	*r* ^a^	–0.13	–0.090	–0.057
	*P* value	.03^b^	13	.34
**Sex, mean (SD)**				
	Female	1.01 (0.83)	3.32 (1.97)	3.92 (1.20)
	Male	1.45 (1.07)	3.91 (1.97)	4.12 (1.26)
	*P* value	.01^b,c^	.05^b^	.31
**Education, mean (SD)**				
	HS^d^ or lower	1.09 (0.92)	3.00 (1.92)	3.79 (1.21)
	Some college (no degree)	1.03 (0.84)	3.21 (1.83)	3.91 (1.14)
	Lower than a college degree	1.15 (0.93)	3.82 (2.02)	4.10 (1.24)
	*P* value	.70	.01^b^	.19
**Hispanic, mean (SD)**				
	Yes	1.31 (1.01)	3.72 (1.79)	4.09 (1.17)
	No	1.04 (0.86)	3.35 (2.03)	3.93 (1.22)
	*P* value	.03^b^	.16	.33
**Language spoken at home, mean (SD)**				
	English	1.10 (0.88)	3.46 (2.0)	3.94 (1.21)
	Other (Spanish)	1.14 (1.03)	3.34 (1.88)	4.10 (1.23)
	*P* value	.76	.72	.47
**Income (US $)** **, mean (SD)**				
	<75,000	1.09 (0.93)	3.16 (1.95)	3.85 (1.24)
	≥75,000	1.12 (0.84)	4.04 (1.92)	4.22 (1.11)
	*P* value	.81	<.001^b^	.001^b^
**Number of children at home, mean (SD)**				
	>1	1.02 (0.82)	3.37 (1.95)	3.97 (1.23)
	1	1.23 (1.01)	3.56 (2.03)	3.96 (1.18)
	*P* value	.06	.42	.91
**Geographic region, mean (SD)**				
	Midwest	1.05 (0.90)	3.15 (2.05)	3.95 (1.06)
	Northeast	1.16 (0.90)	3.75 (1.85)	4.04 (1.37)
	South	1.06 (0.88)	3.39 (2.00)	3.96 (1.19)
	West	1.18 (0.97)	3.55 (1.99)	3.93 (1.25)
	*P* value	.79	.45	.96
**Lost job during the COVID-19 pandemic, mean (SD)**				
	Did not have a job before the COVID-19 pandemic (n=39)	0.68 (0.66)	2.66 (1.70)	4.00 (1.22)
	No (n=158)	1.02 (0.83)	3.37 (1.94)	3.97 (1.23)
	Yes (n=81)	1.46 (1.03)	3.89 (2.05)	3.93 (1.19)
	*P* value	<.001^b^	.01^b^	.95
**Job changed to remote during the COVID-19 pandemic, mean (SD)**				
	No (n=127)	1.02 (0.82)	3.19 (1.92)	4.01 (1.19)
	Yes (n=99)	1.35 (0.96)	4.18 (1.86)	4.06 (1.23)
	*P* value	.01^b^	<.001^b^	.74
**Job hours reduced during the COVID-19 pandemic, mean (SD)**				
	No	1.02 (0.78)	3.45 (2.04)	4.01 (1.14)
	Yes	1.45 (1.04)	3.88 (1.89)	4.00 (1.35)
	*P* value	<.001^b^	.11	.94
**COVID-19’s impact on meeting family needs, mean (SD)**				
	High impact	1.11 (0.99)	3.06 (1.84)	3.6 (1.14)
	Low impact	1.36 (0.87)	3.77 (1.91)	3.95 (1.18)
	Moderate impact	1.14 (0.97)	3.33 (1.92)	4.11 (1.29)
	No impact	0.94 (0.85)	3.38 (2.09)	3.99 (1.21)
	*P* value	.02	.35	.32
**COVID-19’s impact on caregiving hours**				
	*r*	0.09	0.061	0.023
	*P* value	.15	.32	.70
**Change in child screen time since the COVID-19 pandemic**				
	*r*	N/A^e^	0.44	0.15
	*P* value	.02	<.001^b^	.01^b^
**School closed during the COVID-19 pandemic, mean (SD)**				
	Child did not attend school	0.75 (0.78)	2.65 (1.73)	3.93 (1.17)
	No	0.96 (0.84)	3.29 (2.00)	3.69 (1.21)
	Yes	1.40 (0.91)	4.07 (1.93)	4.13 (1.22)
	*P* value	.002^b^	<.001^b^	.06

^a^Pearson correlation coefficient.

^b^*P* values of <.05.

^c^*P* value for independent samples *t* test.

^d^HS: high school.

^e^N/A: not applicable.

### Multivariable Regression Results

In multivariable models (Table 4), having day care, preschool, or school closed during the pandemic and losing a job during the pandemic were most strongly associated with increased child screen time, accounting for 7.23% (*P*<.001) and 3.54% (*P=*.01) of the variance in the outcome after covarying for sociodemographic characteristics. The change in child screen time outcome in turn was most strongly associated with parenting technoference during the pandemic, accounting for 13.5% of the variance (*P*<.001), followed by having day care or preschool or school closed during the pandemic (η^2^=2.40%, *P=*.01) after covarying for sociodemographic characteristics. Parents with a high school education or less reported lower levels of perceived technoference than did parents with a college degree or more (η^2^=2.4%, *P=*.02).

**Table 4:** The results of regression models^a^ (post bivariate screening) by outcome.

				Coefficient		SE		Cohen *d*^b^		*P* value		η^2c^ (%)	
**Change in child screen time (scale 0-4; n=277^d^)**													
	**Sex (reference: female)**												1.93
		Male	0.31		0.13		0.28		.02				
	**Hispanic (reference: no)**												0.16
		Yes	0.08		0.12		0.08		.51				
	Age			–0.01		0.01		–0.32		.01		2.54	
	**Lost job during the pandemic (reference: did not have a job before)**												3.54
		No	0.13		0.16		0.1		.42				
		Yes	0.45		0.17		0.31		.01				
	**School closed during the pandemic (reference: N/A^e^ “child did not attend school”)**												7.23
		No	0.14		0.15		0.12		.33				
		Yes	0.53		0.12		0.52		<.001				
**Technoference (scale 0-6; n=276^d^)**													
	**Sex (reference: female)**												0.11
		Male	–0.15		0.27		–0.07		.59				
	**Education (reference: college degree)**												2.40
		HS^f^ or less	–0.66		0.27		–0.29		.02				
		Some college	–0.48		0.27		–0.21		.08				
	**Lost job during the COVID-19 pandemic (reference: did not have a job before)**												0.60
		No	–0.05		0.34		–0.02		.89				
		Yes	0.27		0.37		0.09		.47				
	**School closed during the COVID-19 pandemic (reference: N/A child did not attend school)**												2.40
		No	0.5		0.31		0.19		.11				
		Yes	0.68		0.27		0.3		.01				
	Change in child screen time since the COVID-19 pandemic			0.82		0.13		0.78		<.001		13.50	
**Parent problem technology use (scale 1-6; n=280)**													
	**Income (reference:<US $75,000)**												2.08
		≥US $75,000	0.37		0.15		0.29		.02				
	Change in child screen time since the COVID-19 pandemic			0.2		0.08		0.31		.01		2.37	

^a^Linear regression model; *P* value for Wald test of significance of regression coefficient.

^b^Cohen *d* effect size interpretation: small=0.2, medium=0.5, and large=0.8 [26].

^c^η^2^ effect size interpretation: small=2%, medium=15%, and large=35% [26].

^d^N<280 due to missing values.

^e^N/A: not applicable.

^f^HS: high school.

## Discussion

### Principal Findings

Serial, national surveys 1 year prior and 1 year into the COVID-19 pandemic revealed increases in child screen time alongside increases in parenting technoference—that is, increases in parents’ perceptions of their own device time interfering with their interactions with their child. Parents reported that their children increased their screen time by approximately 1 hour per day (0.9 hours per day). This finding is consistent with other recent studies [27], including a national survey [3] that found screen time among school-aged children increased by an average of 50 minutes during the early months of the pandemic. The finding of increased parenting technoference is consistent with a recently published study that found an increase in maternal use of mobile devices while parenting during the pandemic lockdowns [16].

We found a strong association between technology use and changes in a child’s schooling, parent work, and remote options for school or work. We also found higher rates of reported technoference among parents with higher levels of educational attainment, as well as among male (vs female) caregivers. These findings are consistent with recent evidence suggesting that increases in child screen time were directly associated with decreased childcare availability [28]. Day care or school closure—which constitutes not just a change in routine but a challenge to supervision requirements for young children, especially if the parent is expected to work while being responsible for children’s needs—was related to increases in parenting technoference as well as child screen time. Another recent study suggested that there was an important interrelationship between parent stressors, the pandemic, and negative parenting techniques such as coercive parenting [29]. Further, there is now a body of literature showing that parenting technoference negatively affects child development through mechanisms such as delayed language acquisition [10,13,18]. The factor most strongly associated with the parenting technoference outcome was the change in child screen time outcome itself. Other studies have shown a strong relationship between parent screen time and child screen time [30,31].

Together these findings suggest that future research and development on interventions designed to mitigate the negative effects of technology on parent-child interactions should consider extrinsic factors and how those may affect the potential feedback loops involved in parent and child device time. These findings may also be critical to informing new consumer health interventions designed for home-based implementation [32,33].

Parent job status was an important contributor to pandemic-related increases in child screen time and technoference. In particular, working parents who experienced job loss during the first year of the pandemic were more likely to report increased child screen time and increased technoference than nonworking parents and parents who did not lose their jobs. Further, working parents (regardless of job loss) were more likely to report technoference than nonworking parents. Several mechanisms may explain these findings. A job-stressed parent may be more likely to use a mobile device as a stress-reduction tool [10,19]. It is also possible that a job-stressed parent is more likely to use a mobile device as a “babysitter” to keep their child occupied as they attend to their own needs, such as job interviews or social support [28]. Future research should explore more deeply the complex interplay between parent and child screen time for working compared to nonworking parents. Program and policy considerations to improve early child development may want to consider different strategies tailored to parent work status.

### Limitations

This study has several limitations common to repeated, cross-sectional observational studies. First, no causal inferences can be made about the timing of the constructs assessed. Experimental studies may better allow for exploring the causal role of how specific contextual events (eg, remote vs in-person work; availability and types of caregivers to supervise children while parents attend to other tasks, child access to nontechnological play materials) affect patterns of parent and child device use, which may be especially relevant in an age of hybrid work policies.

Second, common limitations and biases associated with web-based surveys include social desirability bias and selection bias. Despite oversampling from traditionally underrepresented groups and the matched design to assure greater alignment with the prior survey, these biases may distort the generalizability of these findings. There were inevitably unmeasured confounders omitted from the propensity score matching model. Specifically, this study’s sample included more participants who identified as female, with lower income levels, higher education levels, and a lower prevalence of speaking a language other than English, when compared with the general US population. On the other hand, the results may be generalizable to all 4 census regions of the United States since there was a reasonable representation of respondents from each.

Another limitation was that some items in the prepandemic survey were different, not allowing us to make temporal comparisons. Further, this study was limited by the fact that the measures of parent technoference and child screen time were self-reported and were not externally validated. Parent perceptions of their own technoference and child’s screen time may not correspond to actual levels. Future studies in this area could overcome this limitation by including ecological momentary assessment to capture technoference in real time, or by using newly developing AI-based behavioral observation tools for research. Strategies to mitigate the negative effects of technoference and child screen time ideally should consider both actual and perceived levels of these problems. Finally, it is possible that our specific measures of pandemic stress were not sufficiently sensitive. In this context, it may also be that parent-reported measures of pandemic-related changes in child screen time are better at capturing pandemic-related stress than our direct items about childcare burden.

### Conclusions

Using a national survey representative of all US parents, we found that the COVID-19 pandemic indirectly accelerated preexisting trends of increasing technology and screen use in the lives of young children. In particular, work and day care or school changes due to the COVID-19 pandemic were associated with increased technology interference in the lives of young children. In concert with a growing body of literature, our study further supports the notion that future parenting technoference research and policy making should consider the causes and context behind parenting technoference [34]. In particular, this study suggests important mechanisms through which certain external stresses on parents, especially concerning dual responsibilities of childcare supervision while simultaneously working for pay, may impact healthy child development. For researchers, it motivates the need for more robust studies examining the interrelationships among other contextual factors not examined in this study (eg, availability and types of caregivers, child access to nontechnological play materials), stress, parent screen time, child screen time, and child development. For practitioners, this study buttresses existing recommendations for primary-care providers and childcare educators to guide parents to establish home-based “screen time rules,” not only for their children but also for themselves. To help support families in this effort, our findings add updated context to existing policy recommendations from the American Academy of Pediatrics and other national organizations about the safe use of screens and screen time by young children and their adult caregivers. State and federal policymakers should consider these findings to inform evolving regulations that pertain to child exposure to new media (eg, social media, apps, and virtual-reality headsets), and consider its implications for research funding to strengthen the causal evidence base on the positive and negative impact of these media on parent and child well-being.

## Appendix

Multimedia Appendix 1 Survey administered.

## Abbreviations

N/A: not applicable
