# Supplementary material for: Evidence for Changes in Screen Use in the United States During Early Childhood Related to COVID-19 Pandemic Parent Stressors: Repeated Cross-Sectional Study
Source: JMIR Pediatr Parent. 2024 May 22;7:e43315. doi: 10.2196/43315 (PMC11153974; doi:10.2196/43315)
Supplement: Multimedia Appendix 1 [file pediatrics_v7i1e43315_app1.docx]

**Multimedia Appendix 1.** AI parenting survey COVID-19. Delivered by CloudResearch via Qualtrics.

| **AI Survey Block 2, Q1** | | | Respondents, No. (%) | | | | |  |
| --- | --- | --- | --- | --- | --- | --- | --- | --- |
| The following questions ask about your use of mobile electronic devices. By mobile electronic devices, we mean mobile phones and other devices -- including tablets (e.g., iPad, Kindle Fire, Galaxy Touch) and other handheld devices (e.g., iPod touch, AppleWatch, Kindle, Nintendo Switch).     In a typical day, for about how many HOURS do you use each of the following: | | | | | | | |  |
|  | 0 hours (1) | <1 hour (2) | | 1 hour (3) | 2 hours (4) | 3 hours (5) | 4+ hours (6) | |
| A television (TV) (1) |  |  | |  |  |  |  | |
| A desktop or laptop computer (2) |  |  | |  |  |  |  | |
| A mobile phone without internet connectivity (3) |  |  | |  |  |  |  | |
| A mobile phone with internet connectivity (smartphone) (4) |  |  | |  |  |  |  | |
| A tablet (e.g., iPad, Kindle Fire, Galaxy Touch) (5) |  |  | |  |  |  |  | |
| Other handheld electronic devices (e.g., iPod touch, Apple Watch, Kindle) (6) |  |  | |  |  |  |  | |
| A video game console (7) |  |  | |  |  |  |  | |

| **AI Survey Block 2, Q2** | Respondents No. (%) |
| --- | --- |
| Now think of your youngest child.  How old is your youngest child (years)? | |
| Under 1 (1) |  |
| 1 (6) |  |
| 2 (2) |  |
| 3 (3) |  |
| 4 (4) |  |
| 5 (5) |  |

| **AI Survey Block 2, Q3** | | | Respondents No. (%) | | | |
| --- | --- | --- | --- | --- | --- | --- |
| In a typical day, how many TIMES does each of the following devices interrupt a conversation or activity between you and your child? | | | | | | |
|  | 0 times (1) | 1 time (2) | | 2 times (3) | 3 times (4) | 4+ times (5) |
| A television (TV) (1) |  |  | |  |  |  |
| A desktop or laptop computer (2) |  |  | |  |  |  |
| A mobile phone without internet connectivity (3) |  |  | |  |  |  |
| A mobile phone with internet connectivity (smartphone) (4) |  |  | |  |  |  |
| A tablet (e.g., iPad, Kindle Fire, Galaxy touch) (5) |  |  | |  |  |  |
| Other handheld device (e.g., iPod touch, Apple Watch, Kindle) (6) |  |  | |  |  |  |
| A video game console (7) |  |  | |  |  |  |

| **AI Survey Block 2, Q4** | | | Respondents No. (%) | | |
| --- | --- | --- | --- | --- | --- |
| Think about the last few times a device interrupted a conversation or activity between you and **your youngest child**. What was your child doing? | | | | | |
|  | Never (1) | Sometimes (3) | | Most of the time (4) | Almost all the time (5) |
| Reading (1) |  |  | |  |  |
| Playing (2) |  |  | |  |  |
| Eating (3) |  |  | |  |  |
| Cooking together (4) |  |  | |  |  |
| Coloring/Drawing (5) |  |  | |  |  |
| Bath time (6) |  |  | |  |  |
| Getting dressed (7) |  |  | |  |  |
| Having a conversation with me (8) |  |  | |  |  |

| **AI Survey Block 2, Q5** | | | Respondents No. (%) | | |
| --- | --- | --- | --- | --- | --- |
| Where are you and **your youngest child** usually located when a device interrupts a conversation or activity between the two of you? | | | | | |
|  | Never (1) | Sometimes (2) | | Most of the time (3) | Almost all the time (4) |
| In the living room (1) |  |  | |  |  |
| In the bedroom (2) |  |  | |  |  |
| In the kitchen (3) |  |  | |  |  |
| In a home office (4) |  |  | |  |  |

| **AI Survey Block 3, Q6** | | | Respondents No. (%) | | | | |  |
| --- | --- | --- | --- | --- | --- | --- | --- | --- |
| **Please rate your agreement with each statement, using the following scale:** | | | | | | | |  |
|  | Strongly Disagree (57) | Disagree (58) | | Slightly Disagree (59) | Slightly Agree (60) | Agree (61) | Strongly Agree (62) | |
| When my mobile electronic device alerts me to indicate new messages, I cannot resist checking them. (5) |  |  | |  |  |  |  | |
| I often think about calls or messages I might receive on my mobile phone. (6) |  |  | |  |  |  |  | |
| I feel like I use my mobile phone too much. (7) |  |  | |  |  |  |  | |
| I am worried about the impact of my mobile electronic device use on my child. (9) |  |  | |  |  |  |  | |
| I would like help in limiting my mobile electronic device use around my child. (10) |  |  | |  |  |  |  | |
| I have tried to limit my mobile electronic device use around my child. (12) |  |  | |  |  |  |  | |

| **AI Survey Block 3, Q7** | | | Respondents No. (%) | | | | |  |
| --- | --- | --- | --- | --- | --- | --- | --- | --- |
| **Please rate your agreement with each statement, using the following scale:** | | | | | | | |  |
|  | Strongly Disagree (48) | Disagree (49) | | Slightly Disagree (50) | Slightly Agree (51) | Agree (52) | Strongly Agree (53) | |
| To help me use electronic devices effectively in my child’s presence - I would **attend a class.** (2) |  |  | |  |  |  |  | |
| To help me use electronic devices effectively in my child’s presence - I would **attend a support group with other parents.** (3) |  |  | |  |  |  |  | |
| To help me use electronic devices effectively in my child’s presence - I would consider **help from a coach in my home.** (4) |  |  | |  |  |  |  | |
| To help me use electronic devices effectively in my child's presence - I would consider **online or video chat help from a live coach.** (1) |  |  | |  |  |  |  | |

| **AI Survey Block 4, Q8** | | | Respondents No. (%) | | | | |  |
| --- | --- | --- | --- | --- | --- | --- | --- | --- |
| Some electronic devices are currently being designed to HELP you have a better connection with your child – by coaching you or giving you meaningful, real-time feedback.   Imagine such a “computer-assisted coach,” which you could use in your home to get feedback on your use of electronic devices while caring for your child.  The computer-assisted coach would automatically analyze computer vision and other data to provide the feedback. Whenever you want, you could turn this computer-assisted coach on or off.  Please rate your agreement with each statement below, using the following scale: | | | | | | | |  |
|  | Strongly Disagree (43) | Disagree (44) | | Slightly Disagree (45) | Slightly Agree (46) | Agree (47) | Strongly Agree (48) | |
| Using a computer-assisted coach while caring for my child would **help me be more aware of my device use around my child.** (36) |  |  | |  |  |  |  | |
| Using a computer-assisted coach while caring for my child would **improve my interactions with my child.** (37) |  |  | |  |  |  |  | |
| Using a computer-assisted coach while caring for my child would **help me be a better parent.** (38) |  |  | |  |  |  |  | |
| Using a computer-assisted coach while caring for my child would **raise privacy concerns.** (40) |  |  | |  |  |  |  | |
| Using a computer-assisted coach while caring for my child would **help me notice more quickly when my device use is interfering with my caregiving.** (42) |  |  | |  |  |  |  | |
| Using a computer-assisted coach while caring for my child would **help me keep my attention focused on my child.** (35) |  |  | |  |  |  |  | |
| Using a computer-assisted coach while caring for my child would **be useful to me.** (39) |  |  | |  |  |  |  | |

| **AI Survey Block 7, Q10** | Respondents No. (%) |
| --- | --- |
| Which, if any, of the following voice-controlled smart speakers do you have inside your home? (Check all that apply) | |
| Amazon Echo (1) |  |
| Amazon Alexa (5) |  |
| Google Home (6) |  |
| None (7) |  |
| Other (4) |  |

| **AI Survey Block 7, Q9** | Respondents No. (%) |
| --- | --- |
| Which, if any, of the following video monitoring devices do you have inside your home? (Check all that apply) | |
| Google Nest Cam (6) |  |
| Arlo (7) |  |
| Amazon Cloud Cam (8) |  |
| SimpliSafe (9) |  |
| None (11) |  |
| Other (4) |  |

| **AI Survey Block 7, Q34** | Respondents No. (%) |
| --- | --- |
| Please rate your agreement with the following statement:   I would be comfortable sharing secure recordings from voice/video monitoring devices for research purposes. These data would be used only for research purposes at Stanford University, to help support parents and child well-being. | |
| Strongly Disagree (2) |  |
| Disagree (3) |  |
| Slightly Disagree (4) |  |
| Slightly Agree (5) |  |
| Agree (6) |  |
| Strongly Agree (7) |  |

| **AI Survey Block 7, Q66** | Respondents No. (%) |
| --- | --- |
| Now please think about your life over the past year.  The following questions ask about how things have changed as a direct or indirect result of the pandemic.  At any time during the coronavirus (COVID-19) pandemic, did you lose a job? | |
| Yes (1) |  |
| No (2) |  |
| N/A (I did not have a job outside the home before the pandemic) (3) |  |

| **AI Survey Block 7, Q12** | | Respondents No. (%) | |
| --- | --- | --- | --- |
| At any time during the coronavirus (COVID-19) pandemic, | | | |
|  | Yes (1) | No (2) | N/A (3) |
| did your job change to be mainly remotely from home? (6) |  |  |  |
| were the hours of your job reduced below what you like them to be? (1) |  |  |  |
| did you go on furlough from your job? (2) |  |  |  |

| **AI Survey Block 7, Q12** | Respondents No. (%) |
| --- | --- |
| At any time during the coronavirus (COVID-19) pandemic, did **your youngest child’s** daycare, preschool or Kindergarten close? | |
| Yes (1) |  |
| No (2) |  |
| N/A (my youngest child did not attend daycare or school) (3) |  |

| **AI Survey Block 7, Q13** | Respondents No. (%) |
| --- | --- |
| At any time during the coronavirus (COVID-19) pandemic, did **your youngest child** get a new in-home caregiver (e.g., babysitter or nanny) | |
| Yes (1) |  |
| No (2) |  |

| **AI Survey Block 7, Q14** | Respondents No. (%) |
| --- | --- |
| Since the coronavirus (COVID-19) pandemic began, by how many hours per week did you **increase** the time you spend providing child care for **your youngest child**? | |
| I decreased hours (1) |  |
| 0 hours (4) |  |
| [null] (5) |  |
| 1 hour (6) |  |
| 2 hours (7) |  |
| 3 hours (8) |  |
| 4+ (9) |  |

| **AI Survey Block 7, Q15** | Respondents No. (%) |
| --- | --- |
| At any time during the coronavirus (COVID-19) pandemic**,** did **your youngest child** have **another parent** or relative who helps you provide childcare for your youngest child? | |
| Yes, another caregiver (SAME as pre-COVID) (1) |  |
| Yes, another caregiver (DIFFERENT than pre-COVID) (3) |  |
| No, I am the only caregiver (2) |  |

| **AI Survey Block 7, Q16** | Respondents No. (%) |
| --- | --- |
| At any time during the coronavirus (COVID-19) pandemic, by how many hours per week did **your youngest child**’s other caregiver **increase** their time providing child care for your youngest child? | |
| They decreased hours (1) |  |
| 0 hours (6) |  |
| [blank] (7) |  |
| 1 hour (8) |  |
| 2 hours (9) |  |
| 3 hours (10) |  |
| 4+ hours (11) |  |

| **AI Survey Block 7, Q18** | | Respondents No. (%) | |
| --- | --- | --- | --- |
| Since the coronavirus (COVID-19) pandemic began, how difficult is it for you to meet the following needs for your family: | | | |
|  | Less difficult than before (1) | Same as before (3) | More difficult than before (4) |
| See a healthcare provider if you or your family need it (1) |  |  |  |
| Have enough money for food for you and your family? (2) |  |  |  |
| Have enough money to pay for electricity or heating or water? (3) |  |  |  |
| Have enough money to pay for housing? (4) |  |  |  |

| **AI Survey Block 7, Q17** | Respondents No. (%) |
| --- | --- |
| Please tell us other ways in which the COVID-19 pandemic affected your job and childcare situations. | |

| **AI Survey Block 7, Q65** | Respondents No. (%) |
| --- | --- |
| Since the coronavirus (COVID-19) pandemic began, how difficult has it been for your family to participate in a distance learning (online) school program for your youngest child: | |
| Not difficult (1) |  |
| Somewhat difficult (2) |  |
| Very difficult (3) |  |
| (N/A) My chid attended in-person school (4) |  |
| (N/A) My child’s school did not have a distance learning option (5) |  |

| **AI Survey Block 7, Q19** | | | Respondents No. (%) | | | |
| --- | --- | --- | --- | --- | --- | --- |
| Since the coronavirus (COVID-19) pandemic began, in a typical day, for about how many **MORE HOURS** did **your youngest child** use each of the following? | | | | | | |
|  | 0 more hours (1) | 1 more hour (5) | | 2 more hours (6) | 3 more hours (7) | 4+ more hours (8) |
| A television (TV) (1) |  |  | |  |  |  |
| A desktop or laptop computer (4) |  |  | |  |  |  |
| A mobile phone without internet connectivity (5) |  |  | |  |  |  |
| A mobile phone with internet connectivity (smartphone) (6) |  |  | |  |  |  |
| A tablet (e.g., iPad, Kindle Fire, Galaxy Touch) (7) |  |  | |  |  |  |
| Other handheld electronic devices (e.g., iPod touch, Apple Watch, Kindle) (9) |  |  | |  |  |  |
| A video game console (24) |  |  | |  |  |  |

| **AI Survey Block 7, Q20** | | Respondents No. (%) | | | | |  |
| --- | --- | --- | --- | --- | --- | --- | --- |
| Since the coronavirus (COVID-19). how much of the time is **your youngest child** doing the following electronic activities? | | | | | | |  |
|  | Never (1) | | | Sometimes (2) | Most of the time (3) | Almost all the time (4) | |
| Interactive games and applications (1) |  | | |  |  |  | |
| Watching videos (4) |  | | |  |  |  | |
| Educational apps (5) |  | | |  |  |  | |
| Video chatting with friends/relatives (e.g., Facetime) (6) |  | | |  |  |  | |
| **AI Survey Block 6, Q21** | | | Respondents No. (%) | | | |  |
| Finally, some brief questions about you and your family.  What is your age? | | | | | | |  |
|  | | | | | | |  |

| **AI Survey Block 6, Q22** | Respondents No. (%) |
| --- | --- |
| What is your sex? | |
| Male (1) |  |
| Female (2) |  |
| Other (4) |  |

| **AI Survey Block 6, Q23** | Respondents No. (%) |
| --- | --- |
| Do you consider yourself to be Hispanic or Latino/a/x? | |
| Yes (1) |  |
| No (2) |  |

| **AI Survey Block 6, Q24** | Respondents No. (%) |
| --- | --- |
| What race do you consider yourself to be? | |
| White or Caucasian (2) |  |
| Black or African American (3) |  |
| Asian (5) |  |
| Native Hawaiian or Pacific Islander (6) |  |
| American Indian or Alaska Native (7) |  |
| Other (8) |  |

| **AI Survey Block 6, Q25** | Respondents No. (%) |
| --- | --- |
| How well do you speak English? Would you say..? | |
| Very well (1) |  |
| Well (2) |  |
| Not well (3) |  |
| Not at all (4) |  |

| **AI Survey Block 6, Q26** | Respondents No. (%) |
| --- | --- |
| What language do you usually speak at home? | |
| English (1) |  |
| Spanish (2) |  |
| Other (3) |  |

| **AI Survey Block 6, Q27** | Respondents No. (%) |
| --- | --- |
| How long have you lived in the United States? | |
| I was born in the U.S. (1) |  |
| Less than 1 year (2) |  |
| 1-2 years (3) |  |
| 2-5 years (4) |  |
| More than 5 years (5) |  |

| **AI Survey Block 6, Q28** | Respondents No. (%) |
| --- | --- |
| How would you describe your marital status? | |
| Single, never married (1) |  |
| Living with a partner (2) |  |
| Married (3) |  |
| Separated (4) |  |
| Divorced (5) |  |
| Widowed (6) |  |
| Other (7) |  |

| **AI Survey Block 7, Q29** | Respondents No. (%) |
| --- | --- |
| Including yourself, how many people live in your home? | |
| 1 (71) |  |
| 2 (72) |  |
| 3 (73) |  |
| 4 (74) |  |
| 5 (75) |  |
| 6 (76) |  |
| 7 (77) |  |
| 8 (78) |  |
| 9 (79) |  |
| 10 (80) |  |
| 11+ (81) |  |

| **AI Survey Block 7, Q30** | Respondents No. (%) |
| --- | --- |
| How many children do you have? | |
| 1 (7) |  |
| 2 (8) |  |
| 3 (9) |  |
| 4 (10) |  |
| 5 (11) |  |
| 6 (12) |  |
| 7 (13) |  |
| 8 (14) |  |
| 9 (15) |  |
| 10 (16) |  |
| 11+ (17) |  |

| **AI Survey Block 7, Q31** | Respondents No. (%) |
| --- | --- |
| What is the highest level of school you have completed or the highest degree you have received? | |
| Less than high school degree (1) |  |
| High school degree or equivalent (e.g., GED) (2) |  |
| Some college but no degree (3) |  |
| Associate's degree (4) |  |
| Bachelor's degree (5) |  |
| Graduate degree (6) |  |

| **AI Survey Block 7, Q32** | Respondents No. (%) |
| --- | --- |
| Please mark the group that is closest to your total household income in the past 12 months. | |
| Less than $25,000 (1) |  |
| Between $25,000 and $49,999 (2) |  |
| Between $50,000 and $74,999 (3) |  |
| Between $75,000 and $99,999 (4) |  |
| Between $100,000 and $124,999 (5) |  |
| Between $125,000 and $149,999 (6) |  |
| Between $150,000 and $174,999 (7) |  |
| Between $175,000 and $199,999 (8) |  |
| More than $200,000 (9) |  |
